# Discovery of sporogeny in the malaria transmission-blocking symbiont of *Anopheles* mosquitoes

**DOI:** 10.1128/msphere.00325-25

**Published:** 2025-08-15

**Authors:** Jeremy K. Herren

**Affiliations:** 1 International Centre of Insect Physiology and Ecology (ICIPE) 54662, Nairobi, Kenya; University of Michigan Medical School, Ann Arbor, Michigan, USA

**Keywords:** *Microsporidia*, symbiont, malaria control, *Anopheles coluzzii*, vertical transmission, octosporogony, host–symbiont interactions

## Abstract

In recent work, Parry et al. provide a detailed visualization of *Microsporidia* MB sporogeny (E. R. S. Parry, R. Pevsner, B. C. Poulton, D.-K. Purusothaman, et al., mSphere 10:e00851-24, 2025, https://doi.org/10.1128/msphere.00851-24). Their findings reveal that *Microsporidia* MB is localized to mosquito germline tissues and undergoes octosporogony within developing oocytes, suggesting both vertical and potential horizontal transmission routes. The identification of spores with hallmark microsporidian structures strengthens the case for environmental dissemination via egg deposition. These results fill key biological knowledge gaps and set new methodological standards for *in vivo* symbiont imaging. This work advances *Microsporidia* MB’s feasibility as a transmission-blocking symbiont and supports its development as a novel, sustainable malaria control tool.

## COMMENTARY

In recent work, Parry et al. provide the first detailed description of *Microsporidia* MB’s lifecycle in *Anopheles coluzzii* using advanced confocal and electron microscopy techniques (5). *Microsporidia* MB blocks *Plasmodium falciparum* transmission in *Anopheles arabiensis* (1) and therefore has promise as a novel biological control agent (2). Despite applied significance, many aspects of the biology and transmission modes of *Microsporidia* MB are unknown. This paper not only fills critical knowledge gaps about *Microsporidia* MB’s development and transmission but also establishes important methods to visualize and quantify the *Anopheles* symbiont. In addition, Parry et al. provide the first visual characterization of *Microsporidia* sp. MB in *A. coluzzii,* advancing the prospect of developing a *Plasmodium* transmission blocking strategy encompassing diverse *Anopheles* mosquito vectors.

A key takeaway from this study is the observation that *Microsporidia* MB is localized to mosquito germline tissues, specifically ovaries in females, testes in males, and gonadal imaginal discs in larvae. While previous studies did indicate some of these were the primary sites of infection (3, 4), it was previously not possible to exclude the presence of *Microsporidia* MB infection in other tissues. Parry et al. use FISH and paraffin sectioning, which enables the authors to examine whole *Anopheles* mosquitoes and to determine the pattern of *Microsporidia* MB distribution. The extent to which *Microsporidia* MB infection is specific to gonads is in line with a highly specialized symbiosis, exhibiting adaptations for efficient vertical transmission and minimization of host fitness costs. The imaging methodologies used—fluorescence *in situ* hybridization (FISH), paraffin sectioning, and transmission election microscopy (TEM)—also contribute methodologically to the field. They set a new benchmark for investigating the localization and developmental staging of symbionts *in vivo*.

The identification of sporogony, particularly in the “light” phenotype oocytes characterized by depleted yolk and high spore density, suggests a potential horizontal transmission route via egg deposition in larval habitats. Parry et al. show that *Microsporidia *MB undergoes octosporogony—a form of sporulation that produces eight spores within a sporophorous vacuole—inside developing oocytes. These spores were shown to possess characteristic microsporidian structures like the polar filament and chitinous spore wall through TEM, strengthening the case for their infectivity. These findings add a new dimension to the understanding of *Microsporidia* MB’s transmission ecology: it is not only vertically transmitted but also has a spore-driven component, expanding the avenues for field applications beyond adult mosquito releases. This finding prompts several key questions that are directly influencing our current research in this field. For example, what can *Microsporidia* MB spores infect—could spores introduced into breeding sites colonize larvae and establish transstadial transmission? Are there cues that trigger *Microsporidia* MB sporogony? and can these be harnessed to increase spore production? Clearly, the scope for using *Microsporidia* MB as part of a malaria control strategy will be greatly advanced if researchers can establish an efficient and scalable method to generate infectious *Microsporidia* MB spores.

Parry et al. find that *Microsporidia* MB was not generally present in the *Anopheles* midgut, suggesting a more systemic basis for the *Plasmodium* transmission-blocking phenotype conferred by *Microsporidia* MB in *Anopheles* that is currently under investigation. The discovery that similar *Microsporidia* MB cells could be found in the male testis and female spermathecae will be a solid foundation for additional studies to investigate what happens to sexually acquired *Microsporidia* MB—are they ultimately able to infect the female germline stem cells and ovarioles, or do they directly infect a female’s progeny, or neither? While vertical transmission ensures intergenerational stability, the possibility of environmental dissemination through spores could allow for broader spread without costly mosquito releases. Understanding the full suite of *Microsporidia* MB transmission routes—particularly environmental spore transmission—will inform the design of a possible *Microsporidia* MB-based malaria control intervention.
